# Supplementary material for: Medusa: Software to build and analyze ensembles of genome-scale metabolic network reconstructions
Source: PLoS Comput Biol. 2020 Apr 29;16(4):e1007847. doi: 10.1371/journal.pcbi.1007847 (PMC7213742; doi:10.1371/journal.pcbi.1007847)
Supplement: Attachment — Submitted filename: medusa_reviews.docx [file pcbi.1007847.s001.docx]

Below, our responses to reviewer comments are in bold.

**Comments to the Authors:**

Reviewer #1: This software paper describes the Medusa tool, a software to build and analyse ensembles of genome-scale metabolic network reconstructions. The main interest of Medusa is to enable the simultaneous run and comparison of analyses over families of metabolic network, such as FBA, FVA and gene essentiality. To that goal, a family of metabolic networks is represented in CobraPy Python package using a so-called Ensemble class, which references both a CobrayPy Model (describing the base model) and Features and Members to describes the specificities of the family of metabolic networks. This smart representation allow comparing ensemble of simulations and running pipelines which alternate analyses of families of metabolic networks, then selection of sub-families, followed by novel analysis. An example of such a pipeline is a curation procedure based on the prioritizing of components which contribute the most to simulation uncertainty. Another example is the automatic generation of a family of gap-filled metabolic models according to different media conditions allowing the user to see the impact of the order of the selected media conditions over the gapfilling procedure.

As stated by the author, this representation of families of metabolic networks which relevant classes in CobraPy is a very smart solution to run multiple analyses and workflows and study the variability of metabolic networks while avoiding to store the multiple networks generated along the pipelines. This is particularly useful for automatic analyses involving the generation of multiple models.

However, the paper is lacking some elements to ensure that the Medusa package can be used by the community. They correspond to the four main limitations below that needs to be clarified.

(1) There is no information about the gains enabled by Medusa. What is the maximum number of metabolic networks that an Ensemble can describe ? What is the gain of storage space enabled by the ENSEMBLE representation with respect to a usual representation with individual models ? What are the gains of performance for FBA, FVA, and gene essentiality computations with respect to simulations with individual models ?

**We appreciate these concerns about benchmarking which were also raised by other reviewers--we have added runtime benchmarking for ensemble FBA and ensemble construction via iterative gapfilling to the Medusa documentation. Additionally, we have included an analysis of memory usage and disk storage, comparing an ensemble of models to individual models. All of this benchmarking, and associated tutorial-like text, has been added to the Medusa documentation. We have included figures for gapfilling runtime and ensemble FBA runtime in the manuscript, with references in the text to the rest of the benchmarking results. Below, we summarize these results.**

**We conducted benchmarking to assess both how much RAM is required to load ensembles compared to individual models and how much disk space is required to save files of ensembles compared to individual models. Our calculations indicate that 1000 individual models require about 300 times as much RAM compared to a 1000 member ensemble. Additionally, the compressed saved files for 1000 individual models occupy roughly 175 times as much space as a 1000 member ensemble. These savings are very meaningful, as the memory requirements we calculated for 1000 individual models exceed 16GB (in contrast to a ~50MB ensemble), making it impractical to perform analysis with even a single ensemble with most modern, high performance laptops. The absolute disk space would also be very substantial (~1GB per 1000 models vs. ~6MB for an ensemble).**

**Using a single processor, It takes 2-3 times longer to run FBA on a 1000 member ensemble than to optimize 1000 individual models that have already been pre-solved; however, when the solver is completely reset, an ensemble takes half the time as individual models. (Background: pre-solving occurs when a freshly loaded model is optimized for the first time in COBRApy). When additional processors are used, FBA runtimes in Medusa are below that of individual models, even when the models are pre-solved. We think this is an impressive gain in performance considering that this runtime analysis does not factor in any of the other steps that would be repeated 1000x with individual models during a normal analysis that would not need to be repeated if using Medusa (e.g., changing media conditions).**

**The number of networks is only limited by memory on the user’s computer. We have constructed ensembles with 1000-5000 members in the past with no issues.**

(2) Few details are given about the generation of ENSEMBLE. How can an ENSEMBLE be generated from a family of SBML files generated with other tools ? Does the package enable this ? In addition, the author suggest to store share ENSEMBLES by using a SBML file for the base_model and then to serialize the ENSEMBLE. What is the gain if this is the recommended solution ?

**We have added a section describing how an ensemble can be constructed from a collection of SBML files or COBRApy models stored as json files, as well as a collection of COBRApy models loaded into memory. This section also describes the memory/runtime tradeoffs inherent to these methods, and the flexibility afforded to users.**

**Currently, there is no portion of the SBML specification that would support an ensemble model. We are working towards making this more feasible (e.g., we implemented support for the SBML FBC Groups attribute in cobrapy; this attribute would allow storage of some ensembles in SBML). In the interim, we recommend that users share the base_model for their ensemble as an SBML file to take advantage of SBML interoperability and to preserve some portions of the model that future version of cobrapy might not support. This SBML file, along with the serialized ensemble, should enable future researchers to utilize the ensemble even if significant software rot occurs for Medusa, COBRApy, and/or SBML in the future.**

(3) Figure 4 is quite misleading. What is the advantage of generating Ensembles of gapfilled GENREs by shuffling the different growth conditions ? May the author show FBA and FVA simulations evidencing that the study of the Ensemble generates gains in the study of the considered metabolic network ?

**Figure 4 described the method implemented in Medusa for iterative gapfilling to generate an ensemble. A few principles related to the solutions generated and the performance of the resulting models have been explored in a previous paper by our group which we will reference in this reply: “Managing uncertainty in metabolic network structure and improving predictions using EnsembleFBA”, Biggs & Papin, PLOS Comp Bio 2017. We reference this paper and the key methodology in the introduction of this manuscript, so we do not think that revisions to the manuscript based on this point are warranted.**

**First, the advantage of shuffling growth conditions is that it introduces small variation in the gapfill solution that allows us to quickly find alternative gapfill solutions which are nearly equivalently optimal. In Biggs & Papin, we showed that the gapfill solutions generated during this process are equivalent to the solutions generated by the global optimization problem (i.e., gapfill on all media conditions simultaneously) but that the solve time is many orders of magnitude shorter in the iterative gapfilling case (See Fig 3A-C in Biggs & Papin). Second, similar to the use of probability distributions in statistical modeling, there are inherent methodological gains in generating a distribution of predictions for FBA/FVA, rather than a single solution from one model. One example of the technical innovation enabled by this is described in our section “*Coupling ensemble modeling with machine learning*”; this advance in methodology would be impossible without considering multiple solutions to a gapfilling problem. Third, the performance gains, in terms of common model performance metrics, enabled by ensembles generated using this gapfilling method were detailed rigorously in Biggs & Papin for both FBA and gene essentiality predictions (see figures 4-8 in Biggs & Papin).**

(4) In the introduction, the authors advocate that ENSEMBLEs are useful to handle alternative solutions generated by simulations, including infinite number of feasible flux values within the range determined by a FBA solution space. I do not understand how MEDUSA is able to handle this issue ? Is this possible to generate an ENSEMBLE modeling all such GENREs with alternative feasible fluxes and then to study them with MEDUSA ? How can this be done ? If this is not the case, I would rather suggest to remove the paragraph in the introduction to avoid confusion.

**The FBA example in the introduction only introduces the problem of multiple optima in systems biology; we do not claim that ensembles (or Medusa) handle this issue. We explicitly state that FVA and flux sampling are methods that address this specific issue, then explain how multiple optima arise in many other areas of systems biology, what the consequences are, and how ensembles can address these issues for gapfilling scenarios.**

Reviewer #2: The work presented here represents a useful tool for the community. However, there are some points in the manuscript that require clarification.

• In the example provided in the “Coupling ensemble modeling with machine learning,” it is not clear what data the random forest is trained on. Where it says that the “… random forest regressor is used to predict the simulated values …,” are the authors actually training the random forest on those simulations and then going in to the decision trees to determine the most predictive features? If so, this should be clarified. If not, then the information needs to be added.

**We have added a parenthetical to clarify that input to the random forest regressor is binary reaction presence/absence. We hope this addition, along with the expanded graphical explanation of the method in Figure 2D, make this clear.**

• Some sense of the simulation “wall time” would be helpful for various size models/ensembles. Of course, it will also be necessary to provide information regarding hardware used in order for the wall time to have any significance.

**We have added a benchmarking notebook to the Medusa documentation that describes the time required to generate moderately-sized ensembles (ensembles of 5, 25, or 100 members) on a standard modern laptop. The manuscript now includes a summary of this benchmarking in the text and one figure, including the hardware specifications used for testing.**

• One assumes that Medusa is based on Python 3, and it will become obvious once an individual uses the package. However, it would still be worthwhile to mention the version of Python against which this package is built against.

**Support is provided for Python 3.4 and later, and we have added text specifying that our test system runs Python 3.6 and that we plan to expand this to Python 3.7/3.8. We have also added that no support is provided for Python 2.**

Reviewer #3: In this paper, the authors describe and demonstrate Medusa, a (Python) tool for the analysis of ensemble of metabolic networks. While constraint-based modelling methods have gained immense popularity over the last two decades, they are still beset with a key problem of "degeneracy" in solutions. Furthermore, there exists uncertainty in network structure, in terms of the topology, and many gap-filling algorithms exist to fill gaps (identify missing reactions) in metabolic networks. Building on COBRAPy, Medusa can perform ensembleFBA, to address these and other important issues. Medusa also lends itself to applying machine learning tools for improving curation, and the documentation lists an example of this too.

I found the paper to be very well-written and crisp. It gives a very clear overview of the use of Medusa, and the underlying methods.

Minor Revisions:

1. It would be nice to describe an example of how parallelisation affords advantages, and also a brief discussion of how it scales. It would be advantageous to the users to be well aware of the parallel features of Medusa, which will enable them to work with larger numbers of simulations (or larger networks).

**We have included this in our benchmarking per the discussion above**

2. While Figure 3 is very good and explanative, I would find it interesting to look at a scatterplot of difference in biomass growth rates vs. similarity of the reaction networks in the ensemble. That is, for every pair of networks in the ensemble, plot a point corresponding to (d(network1, network2), v_bio1-vbio2). Is there a clear correlation? What would the outliers, if any, mean? For distance, a Jaccard distance could be used.

**Correlation amongst network similarity metrics and predictions is interesting, but our analysis associated with Figure 3B addresses the heart of your critique much more directly using supervised machine learning. In order to perform your proposed analysis, pairwise network similarities would need to be calculated between each ensemble member, then dimensionality reduction (e.g., ordination) would need to be applied to this distance matrix to obtain a value for each member that could be correlated with biomass production (e.g., PCo1 in Principal Coordinate Analysis). Correlation between network structure and biomass production would only tell you that similar networks have similar biomass production rates. The accompanying notebook in the Medusa documentation in which figure 3B is featured uses a single supervised machine learning model to identify the specific components in a network that should be curated to reduce uncertainty in the simulation performed (here, FBA). This supervised approach gets to the heart of your recommendation much more directly than the analysis you proposed. In order to communicate the value of the concept the reviewer is discussing, we have expanded figure 3B (now Figure 2D) by adding a graphical summary of the supervised machine learning-based curation process.**

3. An example (both in the manuscript) and documentation, as to how ensemble performance can be studied by plotting ROC curves in Medusa could be described.

**We thank the reviewer for this recommendation; we are planning on including an ROC-based evaluation of an ensemble in the documentation when we have implemented functions for calculating metrics for an ROC curve from a dataset and an ensemble. Currently, this requires quite a bit of data wrangling and code outside of the current Medusa API (much moreso than gapfilling), so we will wait to include this functionality in the documentation, rather than the manuscript, when we have improved the model performance assessment capabilities of Medusa.**
