# Supplementary material for: Medusa: Software to build and analyze ensembles of genome-scale metabolic network reconstructions
Source: PLoS Comput Biol. 2020 Apr 29;16(4):e1007847. doi: 10.1371/journal.pcbi.1007847 (PMC7213742; doi:10.1371/journal.pcbi.1007847)
Supplement: Attachment — Submitted filename: medusa_reviews.docx [file pcbi.1007847.s002.docx]

Below, our responses to reviewer comments are in bold.

**Comments to the Authors:**

Reviewer #1: The authors have addressed most of my comments. However, they decided to not directly address my comment on the former figure 4 (now figure 3) about the underlying motivations for the choice of the gap-filling method implemented in medusa to generate ensembl. Although I understand their position, I would suggest as a last minor revision to clearly refer to the paper Biggs & Papin, PLOS Comp Bio 2017 when they introduce Figure 3 (paragraph that starts with "Ensembles can also be generated in Medusa by performing gap-filling on an individual GENRE..." to include a summary of the detailed motivation that they provided in the response to reviewed.

**We thank the reviewer for raising this question and have added further clarifying text describing how gap-filled models generated with this iterative method are equivalent to gap-filled models generated with a single simultaneous optimization problem, yet the iterative procedure solves much more quickly.**
